# A Retrospective Analysis of Hyperlipidemia and COVID-19 Outcomes Investigated in a Rural Midwestern Population

**DOI:** 10.7759/cureus.48211

**Published:** 2023-11-03

**Authors:** Rachel Steffes, Sydney Christensen, Laura Schreck, Nova Beyersdorfer, Darrin S Goade, Kerry Johnson, Greg Stahl, Nicole Ford, Robert D Arnce

**Affiliations:** 1 Department of Osteopathic Medicine, Kansas City University, Kansas City, USA; 2 Department of Osteopathic Medicine, Kansas City University, Joplin, USA; 3 Department of Primary Care, College of Medicine, Kansas City University, Joplin, USA; 4 Department of Pharmacy, Freeman Health System, Joplin, USA; 5 Department of Mathematics, Missouri Southern State University, Joplin, USA; 6 College of Medicine, Kansas City University, Joplin, USA

**Keywords:** cardiovascular disease, serum lipid levels, preventative care, missouri, covid-19, coronavirus, cholesterol, midwest, rural, hyperlipidemia

## Abstract

Background

COVID-19 is a respiratory disease caused by SARS-CoV-2, a coronavirus discovered in 2019. Its impact on the world continues to be studied due to the significant death toll of the disease. As the COVID-19 pandemic remains ongoing, examining the association of COVID-19 with comorbidities and resulting mortality is necessary. This study focuses on population health outcomes with COVID-19 infection and hyperlipidemia (total cholesterol greater than or equal to 200 mg/dL) as a comorbidity, including potential associations with age and sex.

Methods

As a retrospective analytical study, patients were divided into three populations based on COVID-19 and/or hyperlipidemia based on the International Classification of Diseases, Tenth Edition (ICD-10) codes reported in the electronic medical record system at Freeman Health System (FHS) in Southwest Missouri from April 1, 2020, to December 31, 2021. Wald’s methods and two sample proportion summary hypotheses with confidence intervals (CIs) were used for comparison. The populations were subdivided and analyzed for age and sex differences.

Results

Patients with both COVID-19 and hyperlipidemia had a higher mortality rate than patients with COVID-19 and without hyperlipidemia and patients with hyperlipidemia and without COVID-19; patients with COVID-19 and without hyperlipidemia had a higher mortality rate than patients with hyperlipidemia and without COVID-19. All comparisons across these populations were statistically significant (p-value < 0.05). While increased age was associated with increased mortality in all groups, sex was not predictive in this regard.

Conclusion

Our study provides insights into variables affecting COVID-19 outcomes in a rural Midwestern population by showing how the comorbidity hyperlipidemia contributes to increased mortality.

## Introduction

The COVID-19 pandemic has impacted healthcare in substantial ways. Nearly 6.5 million people worldwide [1] and over one million Americans [2] have died from COVID-19 from 2020 to 2022. Due to unknown complications and negative outcomes from the virus and a shift in the need for bed space and hospital resources, the pandemic has contributed to reduced access to care for those suffering from other medical conditions [3]. Reducing the mortality associated with COVID-19 could potentially alleviate the burden of the disease on the healthcare system.

Examining common comorbidities associated with COVID-19 deaths is beneficial to determine how to decrease mortality associated with COVID-19. Hyperlipidemia, defined as total blood cholesterol greater than or equal to 200 mg/dL [1], is an extremely common chronic condition in the United States, affecting around 38% of American adults. Increased cholesterol levels put American adults at greater risk for cardiovascular complications [4]. It has been shown that underlying cardiovascular disease is associated with an increased risk of in-hospital death among patients hospitalized with COVID-19 infection [5].

This study examines how hyperlipidemia contributes to the mortality of COVID-19 by analyzing patients with hyperlipidemia who were hospitalized. The aim was to assess the variability in COVID-19 mortality outcomes among patients diagnosed with hyperlipidemia as a comorbidity. This is a worthwhile endeavor as other comorbidities have been shown to affect COVID-19 mortality outcomes [6]. We opted to study patient populations from the cities of Joplin and Neosho, Missouri, at Freeman Health System (FHS), located near the border of Arkansas, Kansas, and Oklahoma. FHS caters to a diverse and rural population due to its close proximity to small, local communities throughout this four-state area. COVID-19 hospitalization rates and deaths are elevated in rural areas as compared to urban ones [7]. Rural areas generally have an increasingly aging population with more underlying medical conditions [8]. In addition to this, hyperlipidemia remains a long-standing issue in rural communities. This can be due in part to barriers including limited access to medical care and a financial burden [9]. As hyperlipidemia is prevalent in this area, it remains a significant consideration when discussing the mortality of COVID-19 in the Midwest.

## Materials and methods

Data collection

Records were extracted from electronic medical records (EMRs) within FHS in Joplin and Neosho, Missouri. Data was de-identified and collected from April 1, 2020, to December 31, 2021. All participants were over the age of 18 years old. The International Classification of Diseases, Tenth Edition (ICD-10) codes used to collect patient diagnosis information were U071 (COVID-19), E782 (mixed hyperlipidemia), E7849 (other hyperlipidemia), and E785 (hyperlipidemia). The initial dataset highlighted all patients with COVID-19 from the given time period, excluding duplicate admissions. The criteria are outlined inFigure 1 and Figure 2.

**Figure 1:**
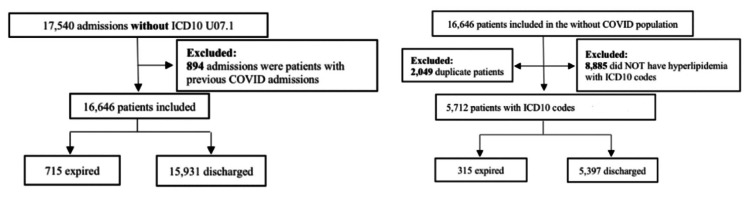
Population Classification Flowchart Without COVID-19 Based on ICD-10 Codes Using ICD-10 codes to collect patient information, the code U071 (COVID-19) was used to exclude those from the sample set. Patients without an ICD-10 code U071 were included and subdivided into expired and discharged populations. The population of patients without COVID-19 was refined based on exclusion criteria, eliminating duplicate patients and patients who did not have hyperlipidemia based on ICD-10 codes E782 (mixed hyperlipidemia), E7849 (other hyperlipidemia), and E785 (hyperlipidemia). These groups were subdivided into patients who expired and those who were discharged. ICD-10: International Classification of Diseases, Tenth Edition, COVID-19: coronavirus disease 2019

**Figure 2:**
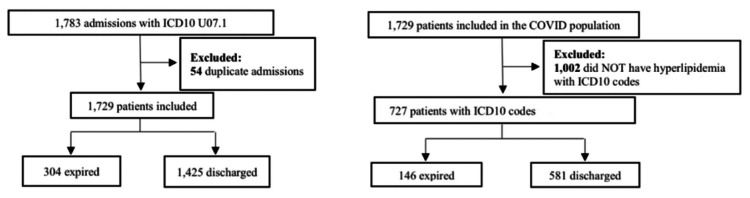
Population Classification Flowchart With COVID-19 Based on ICD-10 Codes The ICD-10 code used to collect patient information was U071 (COVID-19). Populations were excluded based on duplicate admissions and then subdivided into patients who expired and patients who were discharged. The population of COVID-19 patients was subdivided again into those who did and did not have ICD-10 codes for hyperlipidemia as follows: E782 (mixed hyperlipidemia), E7849 (other hyperlipidemia), and E785 (hyperlipidemia). These groups were subdivided into patients who expired and those who were discharged. ICD-10: International Classification of Diseases, Tenth Edition, COVID-19: coronavirus disease 2019

When analyzing age and sex, the populations were redivided into 12 groups and given a population (P) designation. The groups were defined by COVID-19, hyperlipidemia, male, female, greater than or equal to 65 years old, and/or less than 65 years old. Patients who were classified as “less than 65 years old” were 18 years or older. Age and sex data were completed by filtering the raw data for each population. These classifications are shown in Table 1. The datasets analyzed in the current study are available from the corresponding author upon reasonable request.

**Table 1:** Subdivisions of Populations With COVID-19 and Hyperlipidemia Based on Age and Sex Population identifiers based on COVID-19, hyperlipidemia, age, and sex COVID-19: coronavirus disease 2019,

Population Identifier	Patient Characteristics
P1	Males with both COVID-19 and hyperlipidemia
P2	Females with both COVID-19 and hyperlipidemia
P3	Greater than or equal to 65 years old with both COVID-19 and hyperlipidemia
P4	Less than 65 years old with both COVID-19 and hyperlipidemia
P5	Males with COVID-19 and without hyperlipidemia
P6	Females with COVID-19 and without hyperlipidemia
P7	Greater than or equal to 65 years old with COVID-19 and without hyperlipidemia
P8	Less than 65 years old with COVID-19 and without hyperlipidemia
P9	Males with hyperlipidemia and without COVID-19
P10	Females with hyperlipidemia and without COVID-19
P11	Greater than or equal to 65 years old with hyperlipidemia and without COVID-19
P12	Less than 65 years old with hyperlipidemia and without COVID-19

Data analysis

Sample proportions were completed using Wald’s methods. Two-sample proportion summary hypotheses with confidence intervals were calculated for the proportion difference between populations.

## Results

Definition of populations with COVID-19 and hyperlipidemia

**Figure 3:**
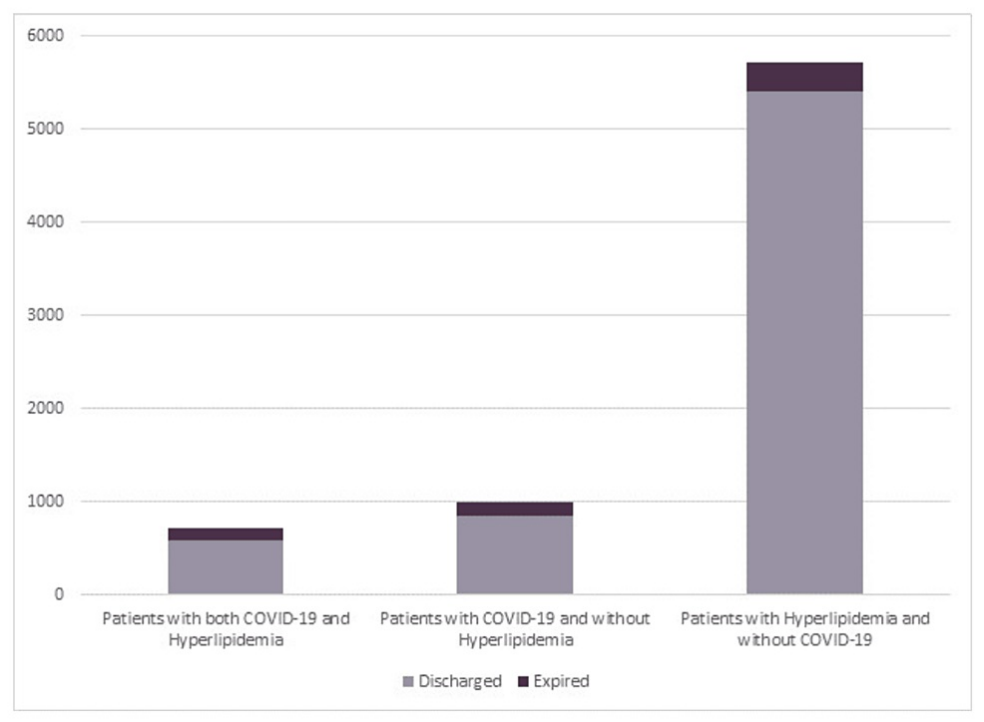
Total Counts of Patient Populations With COVID-19 and Hyperlipidemia Data was extracted from ICD-10 codes from FHS in Joplin and Neosho, Missouri. The population was classified into three groups: patients with both COVID-19 and hyperlipidemia (727 patients in total: 146 expired and 581 discharged) (Figure 1), patients with COVID-19 and without hyperlipidemia (1,002 patients) (Figure 1), and patients with hyperlipidemia and without COVID-19 (5,712 patients in total: 315 expired and 5,397 discharged) (Figure 2). Analysis of intervals was reported with 95% confidence. COVID-19: coronavirus disease 2019, ICD-10: International Classification of Diseases, Tenth Edition

In this retrospective study, data was collected using ICD-10 codes from FHS in Joplin and Neosho, Missouri. The population was classified into three groups: patients with both COVID-19 and hyperlipidemia (727 patients in total: 146 expired and 581 discharged) (Figure 1), patients with COVID-19 and without hyperlipidemia (1,002 patients) (Figure 1), and patients with hyperlipidemia and without COVID-19 (5,712 patients in total: 315 expired and 5,397 discharged) (Figure 2). Data is illustrated in Figure 3. All analysis of intervals was reported with 95% confidence.

Comparison of COVID-19 and hyperlipidemia mortality rates

**Figure 4:**
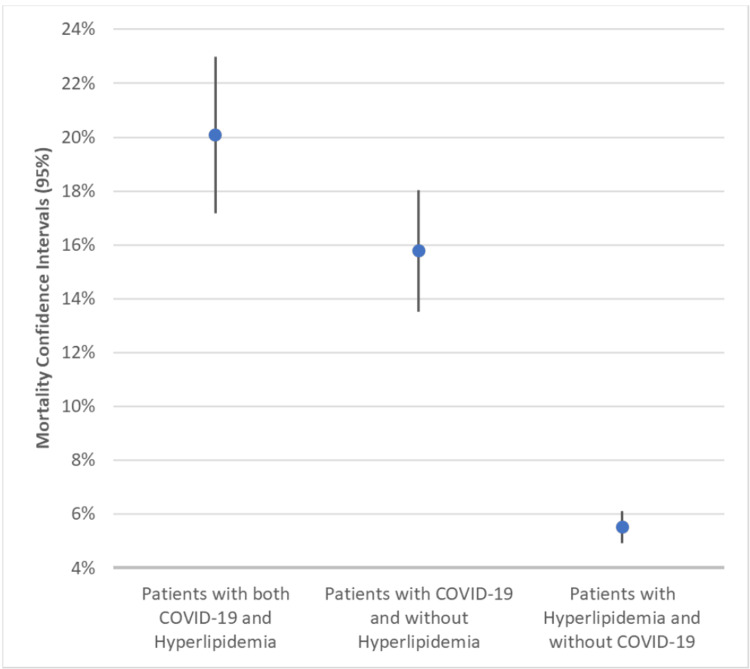
Mortality Confidence Intervals in Individual Populations Based on COVID-19 and Hyperlipidemia Wald’s methods were used for analysis. Populations with and without COVID-19 and/or hyperlipidemia were assessed. Patients with both COVID-19 and hyperlipidemia had a mortality rate between 17.17% and 22.99%. Patients with COVID-19 and without hyperlipidemia had a mortality rate between 13.51% and 18.03%. Patients with hyperlipidemia and without COVID-19 had a mortality rate between 4.92% and 6.11%. COVID-19: coronavirus disease 2019

After an analysis between the initial populations differentiating between COVID-19 and hyperlipidemia, Figure 4shows the confidence intervals (CI) of the mortality rates within each population. Patients with both COVID-19 and hyperlipidemia had a mortality rate between 17.17% and 22.99% (n = 727). Patients with COVID-19 and without hyperlipidemia had a mortality rate between 13.51% and 18.03% (n = 1,002). Patients with hyperlipidemia and without COVID-19 had a mortality rate between 4.92% and 6.11% (n = 5,712). Raw data is outlined in Table 2.

**Table 2:** Mortality of Individual Populations Based on COVID-19 and Hyperlipidemia Raw data outlining the CI for patient populations with COVID-19 and hyperlipidemia. Mortality (with total number) is reflected for each grouping: 146 of 727 patients with both COVID-19 and hyperlipidemia expired, leading to a sample proportion of 0.2008 and thus 20.08% of the population; 158 of 1,002 patients with COVID-19 and without hyperlipidemia expired, creating a sample proportion of 0.1577 and thus 15.77% of the population; and 315 of 5,712 patients with hyperlipidemia and without COVID-19 expired, leading to a sample proportion of 0.0551 and thus 5.51% of the population. CIs (95%) are shown to highlight the range of values within the standard deviation. CI: confidence interval, COVID-19: coronavirus disease 2019

Sample Group	Mortality	Total (Number)	Mortality (p̂) (%)	Standard Error (%)	95% CI (Lower Limit) (%)	95% CI (Upper Limit) (%)
Patients with both COVID-19 and hyperlipidemia	146	727	20.08	1.49	17.17	22.99
Patients with COVID-19 and without hyperlipidemia	158	1,002	15.77	1.15	13.51	18.03
Patients with hyperlipidemia and without COVID-19	315	5,712	5.51	0.30	4.92	6.11

Comparison of COVID-19 and hyperlipidemia mortality rates across populations 

**Figure 5:**
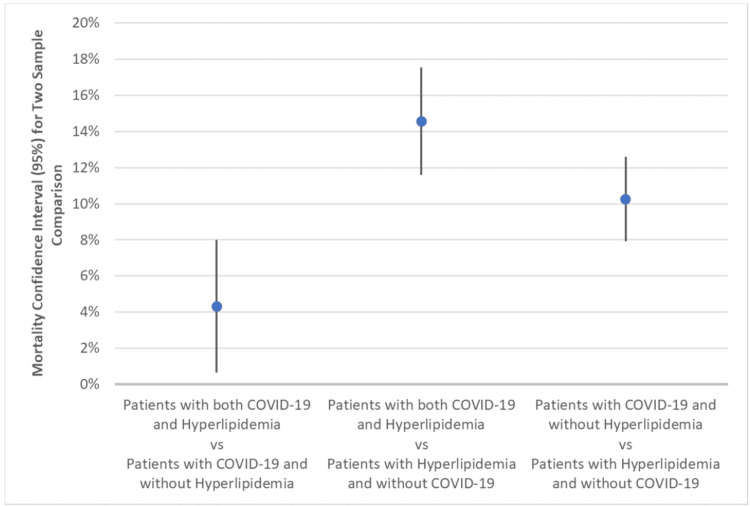
Mortality Confidence Intervals in Two Sample Comparisons Based on COVID-19 and Hyperlipidemia The population of patients with both COVID-19 and hyperlipidemia had a statistically significant mortality rate between 0.63% and 8.00% higher than patients with COVID-19 and without hyperlipidemia (p-value = 0.02). The population of patients with both COVID-19 and hyperlipidemia had a statistically significant mortality rate between 11.60% and 17.54% higher than patients with hyperlipidemia and without COVID-19 (p-value < 0.0001). The population of patients with COVID-19 and without hyperlipidemia had a statistically significant mortality rate between 7.92% and 12.59% higher than the patients with hyperlipidemia and without COVID-19 (p-value < 0.0001). Two sample proportion summary hypotheses with CI were calculated for the proportion difference between populations. CI: confidence interval, COVID-19: coronavirus disease 2019

In comparing populations among each other, Figure 5 shows the CI of mortality rates between populations. Each comparison was found to be statistically significant (p-value < 0.05). The population of patients with both COVID-19 and hyperlipidemia (n = 727) had a mortality rate between 0.63% and 8.00% higher than patients with COVID-19 and without hyperlipidemia (n = 1,002) (p-value = 0.02). The population of patients with both COVID-19 and hyperlipidemia (n = 727) had a mortality rate between 11.60% and 17.54% higher than patients with hyperlipidemia and without COVID-19 (n = 5,712) (p-value < 0.0001). The population of patients with COVID-19 and without hyperlipidemia (n = 1,002) had a mortality rate between 7.92% and 12.59% higher than the patients with hyperlipidemia and without COVID-19 (n = 5,712) (p-value < 0.0001). Raw data is outlined in Table 3.

**Table 3:** Mortality of Two Sample Comparisons Based on COVID-19 and Hyperlipidemia With Confidence Intervals Raw data comparing the patient populations with COVID-19 and hyperlipidemia and CI. Using values obtained from Table 2, mortality (number) is reflected for each grouping: 146 of 727 patients with both COVID-19 and hyperlipidemia expired, with a sample proportion of 0.2008 (20.08% of the population); 158 of 1,002 patients with COVID-19 and without hyperlipidemia expired, with a sample proportion of 0.1577 (15.77% of the population); and 315 of 5,712 patients with hyperlipidemia and without COVID-19 expired, with a sample proportion of 0.0551 (5.51% of the population). The difference was calculated between the two sample proportions of the groups compared, with a 4.31% mortality difference (95% CI: 0.63%-8.00%, p-value = 0.002) between patients with both COVID-19 and hyperlipidemia and patients with COVID-19 and without hyperlipidemia; a 14.57% mortality difference (95% CI: 11.60%-17.54%, p-value < 0.0001) between patients with both COVID-19 and hyperlipidemia and patients with hyperlipidemia and without COVID-19; and a 10.25% mortality difference (95% CI: 7.92%-12.59%, p-value < 0.0001) between patients with COVID-19 and without hyperlipidemia and patients with hyperlipidemia and without COVID-19. CIs (95%) are shown to highlight the range of values within the standard deviation. CI: confidence interval, COVID-19: coronavirus disease 2019

Sample Comparisons: Sample 1 (S1) // Sample 2 (S2)	Mortality S1	Total (Number) S1	Mortality S2	Total (Number) S2	Sample Difference S1-S2 (%)	Standard Error (%)	95% CI (Lower Limit) (%)	95% CI (Upper Limit) (%)	p-value
Patients with COVID-19 and hyperlipidemia // patients with COVID-19 and without hyperlipidemia	146	727	158	1,002	4.31	1.88	0.63	8.00	0.0200
Patients with COVID-19 and Hyperlipidemia // patients with hyperlipidemia and without COVID-19	146	727	315	5,712	14.57	1.52	11.60	17.54	<0.0001
Patients with COVID-19 and without hyperlipidemia // patients with hyperlipidemia and without COVID-19	158	1,002	315	5,712	10.25	1.19	7.92	12.59	<0.0001

Definitions of populations with COVID-19 and hyperlipidemia and based on age and sex

The same de-identified data used above was re-divided into 12 populations, as seen in Table 1. Table 4 outlines the designations of the 12 population groups: patients who were male with both COVID-19 and hyperlipidemia (P1), patients who were female with both COVID-19 and hyperlipidemia (P2), patients who were greater or equal to 65 years old with both COVID-19 and hyperlipidemia (P3), patients who were less than 65 years old with both COVID-19 and hyperlipidemia (P4), patients who were male with COVID-19 and without hyperlipidemia (P5), patients who were female with COVID-19 and without hyperlipidemia (P6), patients who were greater or equal to 65 years old with COVID-19 and without hyperlipidemia (P7), patients who were less than 65 years old with COVID-19 and without hyperlipidemia (P8), patients who were male with hyperlipidemia and without COVID-19 (P9), patients who were female with hyperlipidemia and without COVID-19 (P10), patients who were greater or equal to 65 years old with hyperlipidemia and without COVID-19 (P11), and patients who were less than 65 years old with hyperlipidemia and without COVID-19 (P12). All analysis of intervals was reported with 95% confidence.

**Table 4:** Mortality of Populations Based on COVID-19, Hyperlipidemia, Age, and Sex With Confidence Intervals Raw data outlining the CI for patient populations based on age, sex, COVID-19, and hyperlipidemia. The first column represents the populations defined in Table 1; the second column represents the mortality (number) of that population; the third column represents the sample proportion of mortality, which is converted to a percentage of mortality by multiplying by 100; and the fourth and fifth columns represent the 95% CIs for the given population, highlighting the range of values within the standard deviation. Each population was analyzed individually to calculate mortality rates within that population using 95% CIs, with P1 (n = 92 of 413) having mortality rates between 18.26% and 26.29%, P2 (n = 54 of 314) between 13.02% and 21.37%, P3 (n = 105 of 465) between 18.78% and 26.38%, P4 (n = 41 of 262) between 11.25% and 20.05%, P5 (n = 88 of 535) between 13.31% and 19.59%, P6 (n = 70 of 467) between 11.75% and 18.23%, P7 (n = 86 of 395) between 17.70% and 25.84%, P8 (n = 72 of 607) between 9.29% and 14.43%, P9 (n = 168 of 2,975) between 4.82% and 6.48%, P10 (n = 147 of 2,737) between 4.53% and 6.22%, P11 (n = 243 of 3,689) between 5.79% and 7.39%, and P12 (n = 72 of 2,023) between 2.75% and 4.37%. CI: confidence interval, COVID-19: coronavirus disease 2019

Sample Group	Mortality	Total (Number)	Mortality (p̂) (%)	Standard Error (%)	95% CI (Lower Limit) (%)	95% CI (Upper Limit) (%)
P1	92	413	22.28	2.05	18.26	26.29
P2	54	314	17.20	2.13	13.02	21.37
P3	105	465	22.58	1.94	18.78	26.38
P4	41	262	15.65	2.24	11.25	20.05
P5	88	535	16.45	1.60	13.31	19.59
P6	70	467	14.99	1.65	11.75	18.23
P7	86	395	21.77	2.08	17.70	25.84
P8	72	607	11.86	1.31	9.29	14.43
P9	168	2,975	5.65	0.42	4.82	6.48
P10	147	2,737	5.37	0.43	4.53	6.22
P11	243	3,689	6.59	0.41	5.79	7.39
P12	72	2,023	3.56	0.41	2.75	4.37

Comparison of COVID-19, hyperlipidemia, age, and sex mortality rates

**Figure 6:**
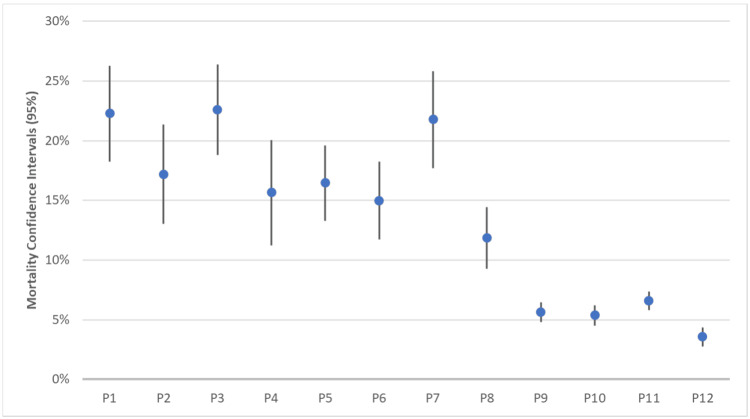
Mortality Confidence Intervals by Individual Populations Divided by Age, Sex, COVID-19, and Hyperlipidemia Using the populations defined in Table 1, raw data in Table 4, and Wald’s methods for analysis, P1 represents patients who were male with both COVID-19 and hyperlipidemia, with a mortality rate between 18.26% and 26.29%; P2 represents patients who were female with both COVID-19 and hyperlipidemia, with a mortality rate between 13.02% and 21.37%; P3 represents patients who were greater or equal to 65 years old with both COVID-19 and hyperlipidemia, with a mortality rate between 18.78% and 26.38%; P4 represents patients who were less than 65 years old with both COVID-19 and hyperlipidemia, with a mortality rate between 11.25% and 20.05%; P5 represents patients who were male with COVID-19 and without hyperlipidemia, with a mortality rate between 13.31% and 19.59%; P6 represents patients who were female with COVID-19 and without hyperlipidemia, with a mortality rate between 11.75% and 18.23%; P7 represents patients who were greater or equal to 65 years old with COVID-19 and without hyperlipidemia, with a mortality rate between 17.70% and 25.84%; P8 represents patients who were less than 65 years old with COVID-19 and without hyperlipidemia, with a mortality rate between 9.29% and 14.43%; P9 represents patients who were male with hyperlipidemia and without COVID-19, with a mortality rate between 4.82% and 6.48%; P10 represents patients who were female with hyperlipidemia and without COVID-19, with a mortality rate between 4.53% and 6.22%; P11 represents patients who were greater or equal to 65 years old with hyperlipidemia and without COVID-19, with a mortality rate between 5.79% and 7.39%; and P12 represents patients who were less than 65 years old with hyperlipidemia and without COVID-19, with a mortality rate between 2.75% and 4.37%. CI: confidence interval, COVID-19: coronavirus disease 2019

Figure 6 represents the CIs of individual populations for mortality based on age and sex as outlined as raw data in Table 4. Each population was analyzed individually to calculate mortality rates within that population, with P1 having mortality rates between 18.26% and 26.29%, P2 between 13.02% and 21.37%, P3 between 18.78% and 26.38%, P4 between 11.25% and 20.05%, P5 between 13.31% and 19.59%, P6 between 11.75% and 18.23%, P7 between 17.70% and 25.84%, P8 between 9.29% and 14.43%, P9 between 4.82% and 6.48%, P10 between 4.53% and 6.22%, P11 between 5.79% and 7.39%, and P12 between 2.75% and 4.37%.

Comparison of COVID-19, hyperlipidemia, age, and sex mortality rates across populations

**Figure 7:**
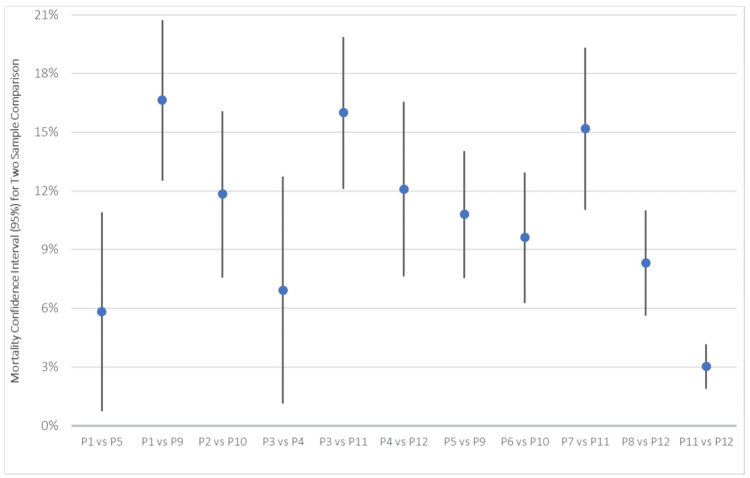
Mortality Confidence Intervals in Two Sample Comparisons Divided by Age, Sex, COVID-19, and Hyperlipidemia The populations were defined in Table 1, with raw data in Table 4, and two sample proportion summary hypotheses with CIs were calculated for the proportion difference between populations. Mortality CIs between the male sex populations were considered statistically significant (p-value < 0.05): P1 and P5, P1 and P9, and P5 and P9. Mortality CIs between female sex populations were considered statistically significant (p-value < 0.05): P2 and P10, and P6 and P10. Mortality CIs between populations with age greater than or equal to 65 years old were considered statistically significant (p-value < 0.05): P3 and P11, and P7 and P11. Mortality CIs between populations with age less than 65 years old were considered statistically significant (p-value < 0.05): P4 and P12, and P8 and P12. In comparisons between populations of differences based on age and sex, the following populations had no statistical significance: P1 and P2 (p-value = 0.0904), P5 and P6 (p-value = 0.5272), and P9 and P10 (p-value = 0.6478). In comparisons between populations of differences based on age and sex, the following populations had statistical significance: P3 and P4 (p-value = 0.0251), with a CI between 1.12% and 12.75%; P7 and P8 (p-value < 0.0001), with a CI between 5.09% and 14.72%; and P11 and P12 (p-value < 0.0001), with a CI between 1.89% and 4.16%. CI: confidence interval, COVID-19: coronavirus disease 2019

Figure 7shows the CIs when comparing different populations against each other as outlined in Table 4. From the previous analysis, in Figure 5, it was found that patient populations with both COVID-19 and hyperlipidemia have a higher mortality rate than either other population, irrespective of age or sex. To confirm this, mortality CIs between the following populations, based on male sex, were considered statistically significant (p-value < 0.05): P1 compared to P5, P1 compared to P9, and P5 compared to P9. Mortality CIs between the following populations, based on female sex, were considered statistically significant (p-value < 0.05): P2 compared to P10 and P6 compared to P10. Mortality CIs between the following populations, based on age greater than or equal to 65 years old, were considered statistically significant (p-value < 0.05): P3 compared to P11 and P7 compared to P11. Mortality CIs between the following populations, based on age less than 65 years old, were considered statistically significant (p-value < 0.05): P4 compared to P12 and P8 compared to P12.

In comparisons between populations of differences based on age and sex, the following populations had no statistical significance: P1 and P2 (p-value = 0.0904), P5 and P6 (p-value = 0.5272), and P9 and P10 (p-value = 0.6478). In comparisons between populations of differences based on age and sex, the following populations had statistical significance: P3 (n = 465) and P4 (n = 262), with a p-value of 0.0251 and CI between 1.12% and 12.75%; P7 (n = 395) and P8 (n = 607), with a p-value of <0.0001 and CI between 5.09% and 14.72%; and P11 (n = 3,689) and P12 (n = 2,023) with a p-value of <0.0001 and CI between 1.89% and 4.16%. Raw data is outlined in Table 5.

**Table 5:** Mortality of Two Sample Comparisons Based on COVID-19, Hyperlipidemia, Age, and Sex With Confidence Intervals Raw data comparing the patient populations with COVID-19 and hyperlipidemia and CI. Sample proportions derived from Table 4. P1 (n = 92 of 413) and P2 (n = 54 of 314) have a difference in sample proportion of 5.08% (p-value = 0.0904). P1 (n = 92 of 413) and P5 (n = 88 of 535) have a difference of 5.83% (p-value = 0.0233). P1 (n = 92 of 413) and P9 (n = 168 of 2,975) have a difference of 16.63% (p-value < 0.0001). P2 (n = 54 of 314) and P6 (n = 70 of 467) have a difference of 2.21% (p-value = 0.4077). P2 (n = 54 of 314) and P10 (n = 147 of 2,737) have a difference of 11.83% (p-value < 0.0001). P3 (n = 105 of 465) and P4 (n = 41 of 262) have a difference of 6.93% (p-value = 0.0251). P3 (n = 105 of 465) and P7 (n = 86 of 395) have a difference of 0.81% (p-value = 0.7762). P3 (n = 105 of 465) and P11 (n = 243 of 3,689) have a difference of 15.99% (p-value < 0.0001). P4 (n = 41 of 262) and P8 (n = 72 of 607) have a difference of 3.79% (p-value = 0.1277). P4 (n = 41 of 262) and P12 (n = 72 of 2,023) have a difference of 12.09% (p-value < 0.0001). P5 (n = 88 of 535) and P6 (n = 70 of 467) have a difference of 1.46% (p-value = 0.5272). P5 (n = 88 of 535) and P9 (n = 168 of 2,975) have a difference of 10.80% (p-value < 0.0001). P6 (n = 70 of 467) and P10 (n = 147 of 2,737) have a difference of 9.62% (p-value < 0.0001). P7 (n = 86 of 395) and P11 (n = 243 of 3,689) have a difference of 15.19% (p-value < 0.0001). P8 (n = 72 of 607) and P12 (n = 72 of 2,023) have a difference of 8.30% (p-value < 0.0001). P11 (n = 243 of 3,689) and P12 (n = 72 of 2,023) have a difference of 3.03% (p-value < 0.0001). P7 (n = 86 of 395) and P8 (n = 72 of 607) have a difference of 9.91% (p-value < 0.0001). P9 (n = 168 of 2,975) and P10 (n = 147 of 2,737) have a difference of 0.28% (p-value = 0.6478). CI: confidence interval, COVID-19: coronavirus disease 2019

Sample Comparisons: Sample 1 (S1) // Sample 2 (S2)	Mortality S1	Total (Number) S1	Mortality S2	Total (Number) S2	Sample Difference: S1-S2 (%)	Standard Error (%)	95% CI (Lower Limit) (%)	95% CI (Upper Limit) (%)	p-value
P1 // P2	92	413	54	314	5.08	2.95	-	-	0.0904
P1 // P5	92	413	88	535	5.83	2.60	0.73	10.92	0.0233
P1 // P9	92	413	168	2,975	16.63	2.09	12.53	20.73	<0.0001
P2 // P6	54	314	70	467	2.21	2.70	-	-	0.4077
P2 // P10	54	314	147	2,737	11.83	2.17	7.57	16.09	<0.0001
P3 // P4	105	465	41	262	6.93	2.97	1.12	12.75	0.0251
P3 // P7	105	465	86	395	0.81	2.84	-	-	0.7762
P3 // P11	105	465	243	3,689	15.99	1.98	12.11	19.88	<0.0001
P4 // P8	41	262	72	607	3.79	2.60	-	-	0.1277
P4 // P12	41	262	72	2,023	12.09	2.28	7.62	16.56	<0.0001
P5 // P6	88	353	70	467	1.46	2.30	-	-	0.5272
P5 // P9	88	535	168	2,975	10.80	1.66	7.55	14.05	<0.0001
P6 // P10	70	467	147	2,737	9.62	1.71	6.27	12.96	<0.0001
P7 // P11	86	395	243	3,689	15.19	2.12	11.04	19.33	<0.0001
P8 // P12	72	607	72	2,023	8.30	1.38	5.61	11.00	<0.0001
P11 // P12	243	3,689	72	2,023	3.03	0.58	1.89	4.16	<0.0001
P7 // P8	86	395	72	607	9.91	2.46	5.09	14.72	<0.0001
P9 // P10	168	2,975	147	2,737	0.28	0.60	-	-	0.6478

## Discussion

In this retrospective analytical study, we assessed the mortality rates of patients in rural communities with COVID-19 and hyperlipidemia and used ICD-10 codes to divide our patient populations. We evaluated our outcomes within three main populations: patients with both COVID-19 and hyperlipidemia (727 patients), patients with COVID-19 and without hyperlipidemia (1,002 patients), and patients with hyperlipidemia and without COVID (5,712 patients). Our results indicate that patient populations with both COVID-19 and hyperlipidemia had significantly higher mortality rates than the population with COVID-19 and hyperlipidemia only irrespective of age or sex.

While patients hospitalized with hyperlipidemia without COVID-19 had mortality rates between 4.92% and 6.11% (n = 5,712) and patients with COVID-19 without hyperlipidemia had mortality rates between 13.51% and 18.03% (n = 1,002), the population with both COVID-19 and hyperlipidemia had significantly higher mortality rates (between 17.17% and 22.99%) (n = 727). The population of patients with both COVID-19 and hyperlipidemia (n = 727) had mortality rates that were 0.63% and 8.00% higher than patients with COVID-19 without hyperlipidemia (n = 1,002) and 11.6% and 17.54% higher than those with hyperlipidemia without COVID-19 (n = 5,712) (p-value < 0.05) (Figure 5). While COVID-19 and hyperlipidemia both increased mortality rates, the combination of both comorbidities led to significantly higher mortality.

This study found that sex was not a statistically significant factor for mortality; however, age was significant in this regard. For this reason, it is essential for clinicians to take age into account when treating a patient greater than 65 years old. The three patient groups with the highest mortality rates (Figure 6) were males with hyperlipidemia and COVID-19 (P1; 18.26%-26.29% mortality rate, n = 413), those equal to or older than 65 years old with hyperlipidemia and COVID-19 (P3; 18.78%-26.38% mortality rate, n = 465), and those equal to or greater than 65 years old with without hyperlipidemia (P7; 17.70%-25.84% mortality rate, n = 395). Notably, females with COVID-19 and hyperlipidemia had the fourth highest mortality rate among the groups (P2; 13.01%-21.32%, n = 314), making it less deadly than the greater than or equal to 65 years old with COVID-19 alone.

The study was limited due to the samples not being randomly selected from the population and therefore may not be representative of the whole. Another limitation was that only one statistical parameter was used when analyzing the results. Due to the novel nature of COVID-19, it would be beneficial to run multiple statistical analyses to ensure no significant factors went undiscovered due to a lack of analysis types. An additional limitation of this study is that it did not take into account the degree of hyperlipidemia within our patients; hyperlipidemia is defined as total blood cholesterol greater than or equal to 200 mg/dL [1]. With the structuring of ICD-10 codes, we were not able to see actual blood work values that led to the diagnosis (and therefore ICD-10 code) of the patient, meaning we do not know by how much their lipid levels were increased above the guidelines. Furthermore, we are unable to know if the patients were being treated for their hyperlipidemia with pharmaceuticals and, if so, how effective their treatment was in reducing their lipid levels [10]. A future direction for this study would be to determine if patients who had well-controlled hyperlipidemia had lower mortality rates than those with untreated or uncontrolled hyperlipidemia. This could feasibly be done via extra data analysis from the patient’s chart as both medications and blood work are available on the EMR.

Another limitation of our study is the time frame for which it took place (April 2020 to December 2021) as COVID-19 cases were happening before and after these studied dates [11]. Additionally, in the early months of the COVID-19 pandemic, tests were limited and often unavailable [12], meaning there is potentially a group of patients who were admitted to the hospital with COVID-19; however, they were not categorized as such in our study. As more research is coming out regarding COVID-19 trends, we could use this information from a similar hospital population and apply it to our data to predict how many of our patients could have potentially been in this category.

Additionally, it should be acknowledged that there were increased rates of skepticism regarding COVID-19 in rural communities [13]. For this reason, it has been documented that patients were not seeking medical attention when they potentially had COVID-19 [14]. This phenomenon could have potentially increased our mortality rates as those who did not seek medical attention were likely not critically ill. While this is a complicated issue to address as it relies on human behavior and beliefs, research could look at patients who have hyperlipidemia and tested positive with outpatient COVID-19 tests and compare them to patients who were hospitalized. This could give insight into hyperlipidemia patients and the severity of COVID-19 impacts.

Lastly, the lifestyle factors that lead to hyperlipidemia are also associated with poor COVID-19 outcomes (obesity, smoking, diet, exercise, etc.) [15], and these things should be taken into account when looking at the mortality rates of hyperlipidemia patients. We were unable to collect this type of data as we performed a retrospective chart review, and this data was not being collected at this time for each patient during their hospitalization; however, future research could examine these factors and the results that they have on patients with COVID-19 and hyperlipidemia [16].

## Conclusions

The role of this study was to determine the impact of hyperlipidemia on COVID-19 mortality outcomes. This study revealed that COVID-19 patients with hyperlipidemia had higher mortality when compared with COVID-19 patients who do not have hyperlipidemia. As much remains unknown about the impacts of exacerbating factors of COVID-19, this information could potentially guide treatment plans for physicians treating COVID-19 patients with hyperlipidemia. Further studies may be done to include a larger geographical area within the United States and could include factors such as age and sex. This could contribute to a broader understanding of the impacts of hyperlipidemia on the mortality of COVID-19 patients across the country.
